# Supplementary material for: Transcriptome Analysis Reveals Vimentin-Induced Disruption of Cell–Cell Associations Augments Breast Cancer Cell Migration
Source: Cells. 2022 Dec 13;11(24):4035. doi: 10.3390/cells11244035 (PMC9776984; doi:10.3390/cells11244035)
Supplement: Supplementary file 1 [file cells-11-04035-s001.zip › DEGs and enrichment analysis.zip!/DEGs and enrichment analysis/WT_VIMvsVector KEGG/ALL/WT_VIMvsVector_ALL.pathway_map.zip!/WT_VIMvsVector_ALL.pathway_map/src/hsa05164.html]

hsa05164


- hsa:4939

- Down regulated genes

ENSG00000111335(-2.07102499896395)

- hsa:3576

- Down regulated genes

ENSG00000169429(-3.18264312774765)

- hsa:4939

- Down regulated genes

ENSG00000111335(-2.07102499896395)

- hsa:91543

- Down regulated genes

ENSG00000134321(-2.15633586706228)

- hsa:56649

- Down regulated genes

ENSG00000137648(-2.71734016077855)

Close
